# Tango therapy for Parkinson's disease: Effects of rush elemental tango therapy

**DOI:** 10.1002/ccr3.2771

**Published:** 2020-04-02

**Authors:** Youngsoon Koh, Geunwoong Noh

**Affiliations:** ^1^ Medical Tango Cheju Halla General Hospital Jeju‐si Korea; ^2^ Department of Allergy and Clinical Immunology Cheju Halla General Hospital Jeju‐si Korea

**Keywords:** dance, dance therapy, old age, Parkinson disease, tango, tango therapy

## Abstract

Tango therapy is very effective by applying tango elements according to the clinical severity and status of a patient. Maximization of the clinical effects of tango therapy in patients with Parkinson's disease may be achieved by the proper prescription of tango elements according to the clinical status of a patient.

## Graphical abstract

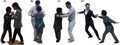

## 1 INTRODUCTION

Tango therapy was conducted for two patients with Parkinson's disease with no dance experience: one with moderate clinical severity and one with severe clinical severity, using the basic elements of Argentine tango systemically for 10 hours in two weeks. Patients improved dramatically with systemic elemental tango therapy for a short duration.

Argentine tango (tango) has been used as a therapy for decades for neurologic disorders, especially for Parkinson's disease (PD).^1^, ^2^ Until now, tango therapy has been conducted only as a dance or a form of exercise.^3^ Most clinical studies have focused on demonstrating the efficacy of tango therapy on PD group without focusing on an appropriate approach according to the clinical status by applying elements of tango movements.^4^

Recently, the necessity of functional anatomical analysis and therapeutic application of tango therapy by the systemic application of the delicate tango elements have been suggested.^5^ The tango terminology is fixed for the systemic description of the therapeutic tango elements.^6^ The basic tango elements are classified for therapeutic purposes.^7^ Thereafter, the posture, stance,^8^ tango gait,^9^ and tango ocho^10^ are analyzed functionally and anatomically.

With this approach, elemental tango therapy is performed by the proper prescription of the basic tango elements according to the patient's clinical severity and status for the first time. Patients with moderate and grave clinical severities of PD were involved. In particular, rush elemental tango therapy was administered for 10 hours over 2 weeks. The patients were notably improved by rush elemental tango therapy for 2 weeks.

## 2 CASE REPORT

### 2.1 Case 1

A 67‐year‐old female patient visited the Department of Rehabilitation Medicine at the Cheju Halla General Hospital due to motor disturbance in all four limbs, gait disturbance, and decreased activities of daily living (ADL) function for 10 years. The diagnosis of PD was made more than 15 years ago in the Department of Neurology of Asan Medical Center (Seoul, Korea). Her mental status was alert. Her cognitive test score is 27 according to the mini‐mental status examination (MMSE).^11^ In the manual muscle test (MMT), both sides were normal. The range of motion (ROM) on both sides was passively full. The sensory test results indicated an intact sensory system. There was no spasticity. The Babinski reflex, ankle clonus, and Hoffmann's sign tests were negative. The activities of daily living (ADL) were totally dependent. The functional level was the level that the patient is able to sit alone. The ambulation was independent when walking with supervision. The clinical course was an insidious onset and slow progression.

**Figure 1:**
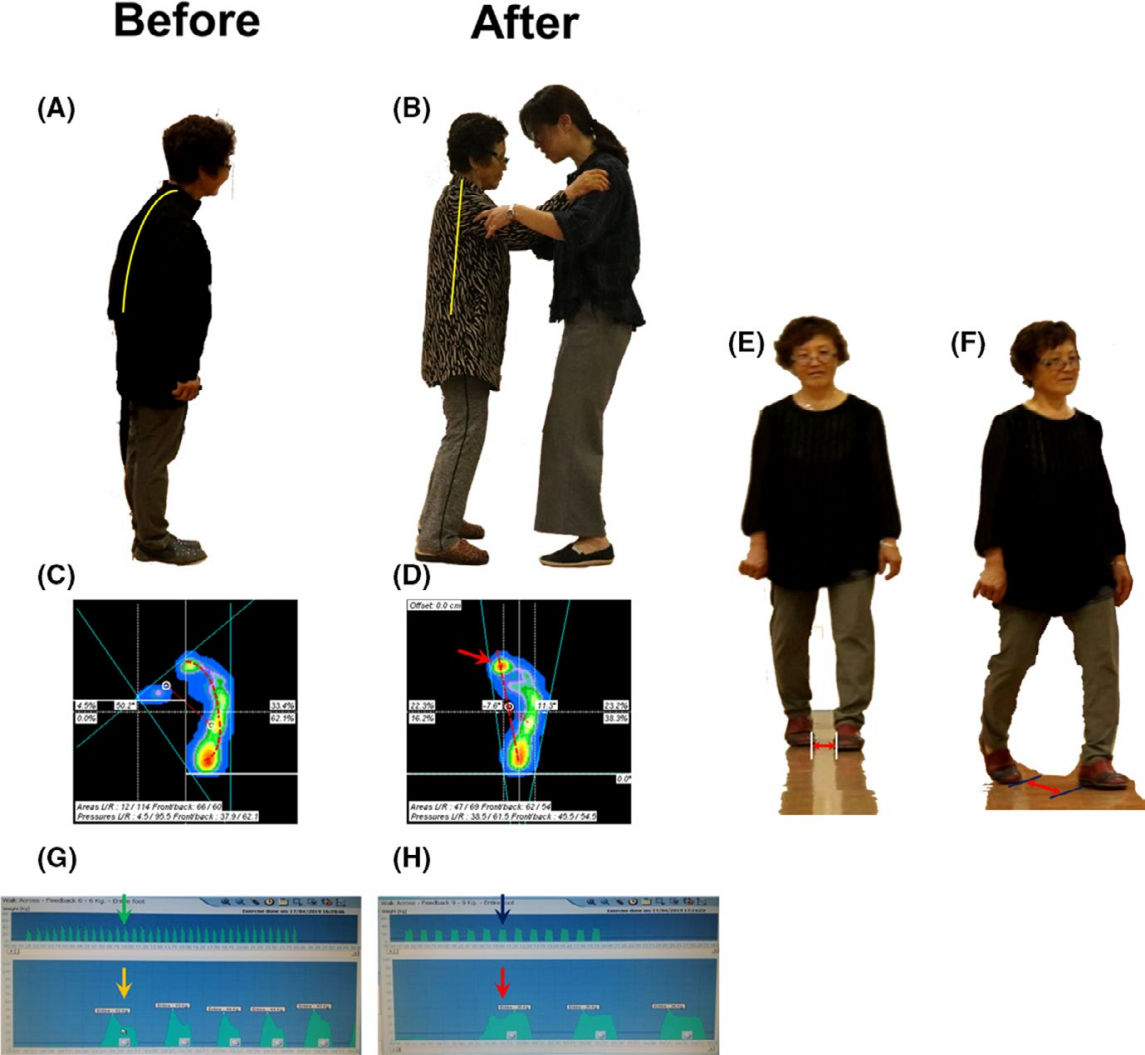
Case 1‐Level 1. The clinical severity of the patient was moderate, and Level 1 tango therapy, including stance, posture, and linear gait, was applied first. A, Before tango therapy, the patient showed a stooped posture. B, Her posture was healthy and upright after tango therapy. C, In the initial evaluation of stepping of the right foot, the body weight was loaded on the lateral arch (red dotted line). D, After tango therapy, the weight‐bearing points of the foot were changed, and the body weight was loaded on the medial arch (red color and red arrow). E, For the initial evaluation of gait, the step width was measured, and she showed a wider step width than the normal (2‐4 cm).^9^ She showed bowed legs. F, As an evaluation of gait, the length of the stride was measured. G, In the gait analysis before tango therapy, she showed rapid speed as a kind of festination (green arrow) with an unstable stepping pattern (yellow arrow). Her freezing of gait was only felt when walking together and holding her elbow, and it was not observed in the gait analysis. H, After tango therapy, her gait speed became normal (black arrow) with a stable stepping pattern (red arrow)

She showed bilateral tremors, bradykinesia, facial masking, and infrequent eye blinking. Additionally, Stellwag's sign test result was positive, and her arm swing was decreased. She also showed cogwheel rigidity, stooped posture (Figure 1A), gait disturbance with freezing and festination, axial instability, and micrographia.

Her clinical status was moderate in clinical severity, and when she started tango therapy, she was taking Levodopa. She fell down frequently in daily life because of impaired balance control during walking, and she felt contractures of all her joints, so she could not sleep with a fully extended, comfortable posture. Her clinical status was staged 3 by the Hoehn & Yahr scale.^12^

For the evaluation of tango therapy, the clinical severities were evaluated by a doctor on a rehabilitation board with the Unified Parkinson's Disease Rating Scale (UPDRS)^13^ and Berg Balance Scale (BBS)^14^ before and after tango therapy. The stepping of the feet and gait was analyzed before and after tango therapy.

For the tango therapy, the initial therapeutic evaluation was performed by a tango therapist according to the basic concepts of the elements of tango.^7^ The therapist analyzed the posture, stance,^8^ and the capability of gait^9^ according to the principles of functional anatomical analysis. Considering the unique gait problems in PD, the gait pattern is also analyzed.

The patient was able to stand independently and stably. Her posture was stooped (Figure 1A). She was able to walk independently but unstably in an unhealthy way. In the evaluation involving holding, the therapist walked together with the patient while holding his or her elbow^6^ and evaluated the patient's level of freezing and festination during gait.

Stance and steps were analyzed using Smart Step (Kwangdeok Medical Co). The device measures the stepping pressure of foot when the patient stands on the sensor board. The stepping analysis revealed that she stood with more pressure on the lateral arches of her feet (Figure 1A). She walked with more pressure on the lateral arches of her feet, bowed thighs and legs, and a slight instability of balance. She felt apparent difficulty in side‐to‐side gait.

Gait was analyzed using Biorescue (RM Ingenirie). The patient wore the shoe, which is equipped with the soft sensor on the inner bottom of shoe to measure stepping pressure, on one foot. The gait analysis showed she had an unstable stepping pattern with freezing and festination. Her step width was very wide; it was more than 10 cm wide during walking (Figure 1E). Additionally, the length of her stride was measured (Figure 1F).

The therapeutic plan for her was a correction of posture, a stepping method, and strengthening of the lower limbs with tango techniques for the improvement and stabilization of balance during walking and the variability of movement with music as cognitive therapy. Most importantly, because she had typical Parkinsonian gait with freezing and festination, the therapist encouraged the patient to adapt her gait to music with a regular rhythm. Thereafter, improvement of her gait by the variation of the rhythm and speed of her gait at her will with music was tried. Namely, the main purpose of tango therapy for this patient was the application of tango gait with music for the improvement of freezing and festination.

**Figure 2:**
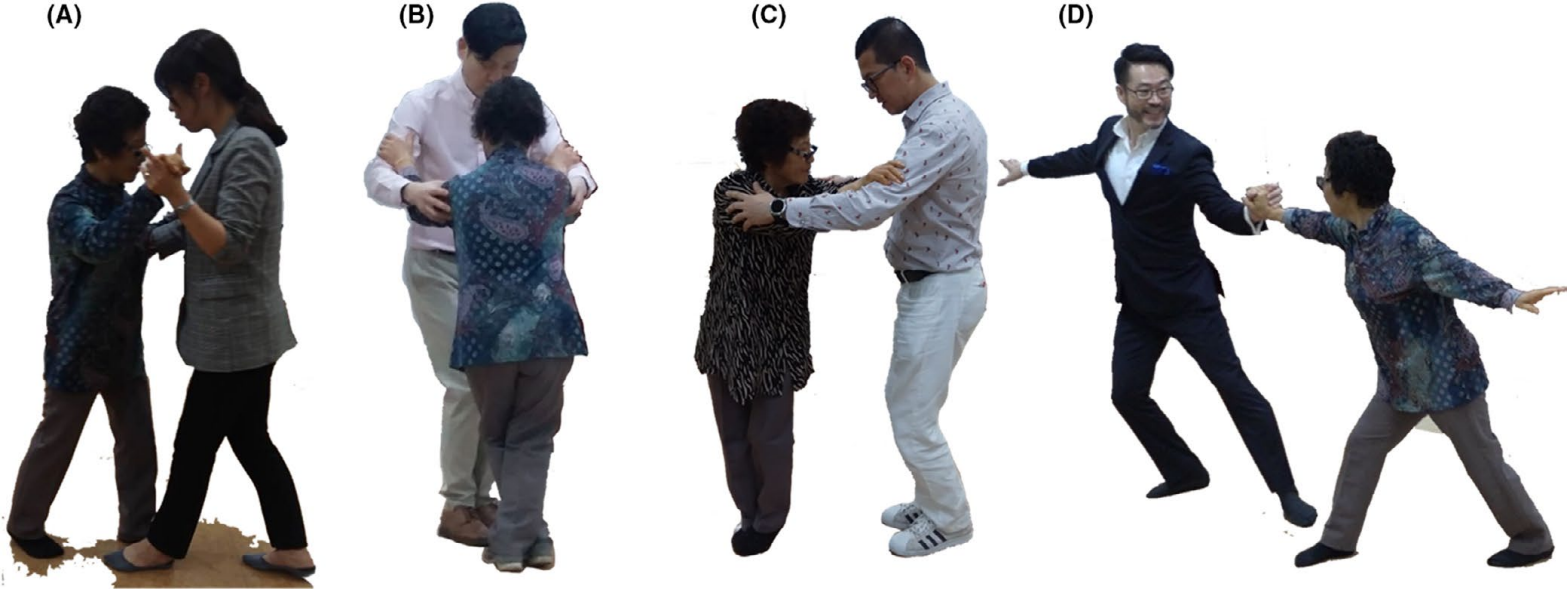
Case 1‐Level 2. After training and the patient was familiar with Level 1 tango therapy, Level 2 tango therapy including cruzada (crossed stance) and pivot (round movement) and Level 3 tango therapy of escenario (choreography) was applied sequentially. A, Before the application of cruzada, the patient was trained to lengthen her stride length to a sufficient length for the crossing of the two feet. B, The cruzada (crossed stance) was tried. C, Pivot was introduced for round movements. D, After the training of cruzada and pivot as the Level 2 tango therapy, choreography (escenario) was introduced as the Level 3 tango therapy

Tango therapy was conducted 1 hour per day, 5 days a week, for two weeks. Tango therapy is performed with one patient alone during a class. As the first step, Level 1 tango therapy was performed, which focuses on stance, posture, and linear gait.^9^ The training in this first step focused on stable linear gait and the lengthening of stride. After Level 1 tango therapy training (Figure 2A), Level 2 tango therapy including cruzada (a stance with both feet crossed) (Figure 2B) and pivot (a round movement in which the body turns on the axis of the weighted limb) (Figure 2C)^10^ was tried. Thereafter, the Level 3 tango therapy of choreography (escenario) was also applied (Figure 2D).

After tango therapy for 10 hours in 2 weeks, the UPDRS score was improved from 32 (grade 3) to 15 (grade 2) points. The improved items were turning in bed and adjusting bed clothes, falling (unrelated to freezing), freezing when walking, walking, rigidity, leg agility, posture, gait, and postural stability in the UPDRS checklist were as expected. These items were expected to be affected by Tango therapy because all these item areas related the main goals of tango therapy. The BBS score was also improved from 32 to 52 points (Table 1A). The patient was stable with medication, without the on‐off phenomena occurring during tango therapy sessions and evaluations of clinical status.

**Table 1:** Clinical severity and severity grade change after tango therapy in patients with a moderate (A) and severe (B) case of Parkinson's disease

	Before	After
A. Case 1. Moderate case		
UPDRS	32 (Grade 3)	15 (Grade 2)
BBS	32	52
B. Case 2. Severe case		
UPDRS	105 (Grade 4)	82 (Grade 3)
BBS	2	12

Note

Patients were dramatically improved after tango therapy for 10 h in 2 wk.

Abbreviations: BBS, Berg Balance Scale^13^; UPDRS, Unified Parkinson's Disease Rating Scale.^12^

Her stepping was improved according to the stepping analysis. The surface pressure on her foot moved from the lateral arch (Figure 1C) to the medial arch of the foot (Figure 1D). Her gait was restored to nearly normal according to the gait analysis (Figure 1G,H). Additionally, her step width returned to normal.

Interestingly, her posture was improved to a healthy upright posture (Figure 1B).

When standing, she stood well balanced with pressure on the medial arches of her feet (Figure 1D). Her gait was straight forward and well balanced. Freezing and festination, which are characteristics of PD, nearly disappeared (Figure 1H). She did not feel the difficulty of walking side‐to‐side with music. Finally, she could dance with music; the freezing gait and festination disappeared, and she moved side‐to‐side freely.

In daily living, falls during walking were nearly absent, and the patient could sleep comfortably with full extension of the body joints. Rush elemental tango therapy was very effective for improving PD symptoms of moderate severity for a short duration of 2 weeks.

### 2.2 Case 2

A 79‐year‐old male patient visited the Department of Rehabilitation Medicine at the Cheju Halla General Hospital for rehabilitation after a rib fracture. The patient showed motor disturbance in all four limbs, gait disturbance, and decreased activities of daily living function for more than 10 years. His cognitive test score is 25 according to the mini‐mental status examination (MMSE). The cognitive function was relatively better compared to the Hoehn and Yahr grade. The range of motion of both sides was passively full. The activities of daily living were nearly totally dependent. The functional level was the level that the patient is able to sit alone. The ambulation was wheelchair‐dependent with propelling ambulation. The clinical course was insidious onset and slow progression. He showed severe bilateral tremors, bradykinesia, facial masking, and infrequent blinking, and his Stellwag's Sign test result was positive. His arm swing was short, and he showed cogwheel rigidity and a stooped posture. Gait disturbances existed with freezing, festination, and very small strides. Additionally, axial instability and micrographia were present. His clinical status was staged 4 by the Hoehn & Yahr scale. The medication he took at the beginning of the tango therapy was a 200 mg dose of Levodopa three times per day. There were no variations in the medication and no on‐off phenomena occurred while conducting tango therapy sessions and evaluations.

**Figure 3:**
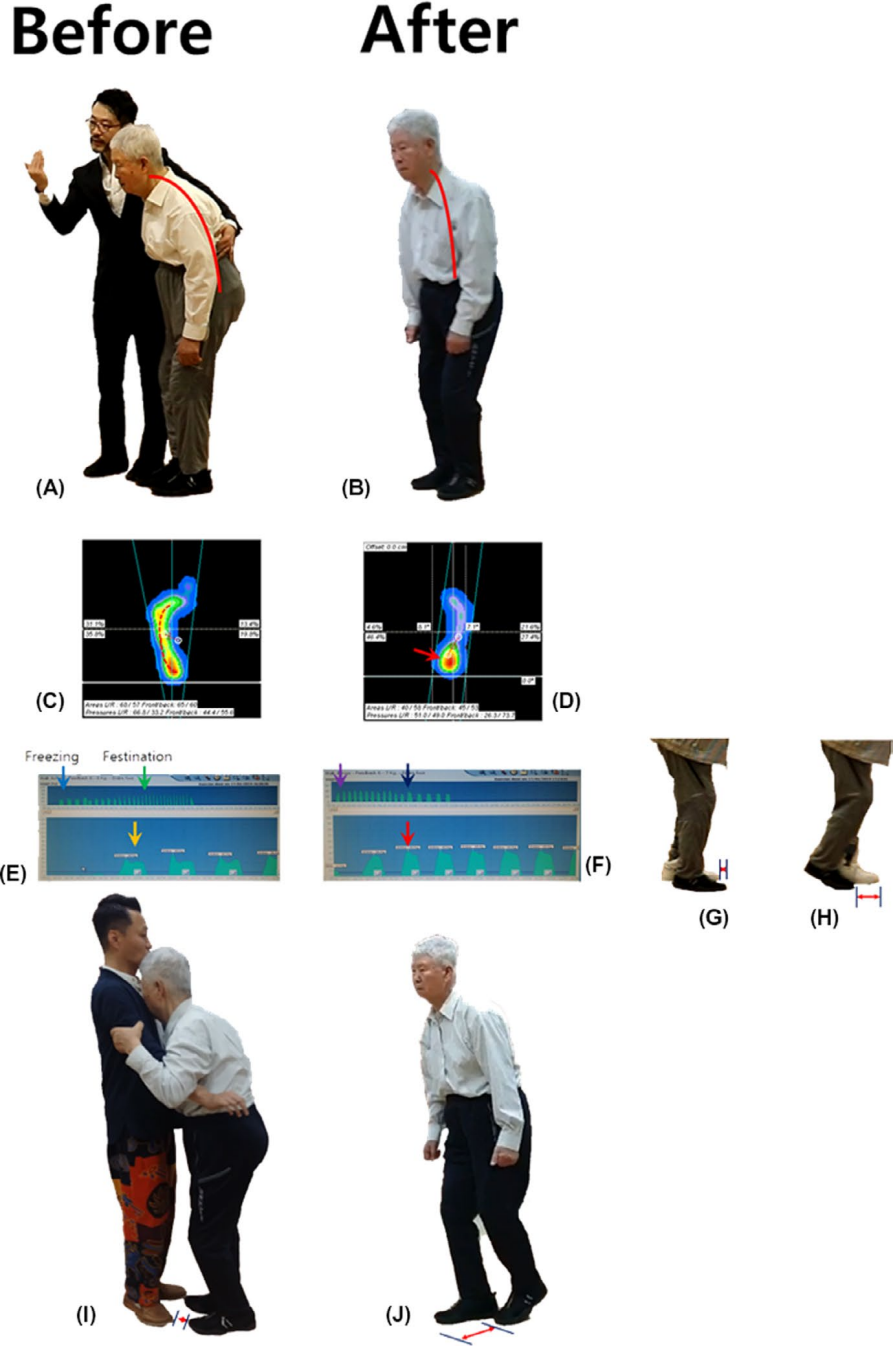
Case 2‐Level 1. The patient's clinical severity was severe. Fortunately, the patient was able to stand with assistance, and Level 1 tango therapy, including stance, posture, and linear gait, was applied as the first step. Before tango therapy, the patient was not able to stand or walk alone (dependent stance and dependent gait), and total therapist‐dependent tango therapy was performed. A, His posture was stooped severely before tango therapy, which created a more unstable stance and gait. B, After tango therapy, his posture was improved dramatically, and even he could stand alone with good posture. C, Before tango therapy, the patient stood bearing his body weight on his lateral arches according to the stepping analysis (red dotted line). D, After tango therapy, he stood bearing his body weight mainly on the area of his heels just below the ankle. The change in the weight‐bearing point seemed to be an effect of the posture correction, although it was not the ideal weight‐bearing point. E, In the gait analysis, the patient showed typical Parkinsonian gait. Freezing gait with initial hesitancy (blue arrow) and festination with the progression of gait (green arrow) was observed before tango therapy. The pressing of the feet on the ground seemed to be stable but heavy and sluggish (yellow arrow). F, After tango therapy, his gait was dramatically improved. Although the hesitancy in the initiation of gait was felt when walking with the patient and holding his elbow, his gait became regular (violet arrow), and festination was not observed (blue arrow). The stepping pattern was stable (red arrow). The gait was asymmetric between the right and left side. The left stride length (G) was longer than the right stride length (H). I, Before tango therapy, he walked totally dependent on the therapist. The right stride length was less than half of the left stride length. J, After tango therapy, the right stride length became longer, nearly the length of the left stride length, even when the patient walked independently without the therapist

The patient was not able to stand independently; when standing alone, he was very unstable, and he was totally dependent on a device (cane) when standing and walking (Figure 3A,I). The posture of the patient was a severely stooped posture (Figure 3A). According to the stepping analysis, he stood with more pressure on the lateral arches of his feet (Figure 3C). He could not walk independently with high stability. Additionally, he walked with more pressure on the lateral arches of his feet, bowed thighs and legs, and a slight instability of balance. Moreover, he had a contracture of the knee joint and hip joint, and he could not stand with a fully extended knee joint or a straight upright posture of the vertebral column (Figure 3I). According to the gait analysis, he showed typical freezing and festination with the progression of walking (Figure 3E). Additionally, he showed resting pill‐rolling tremors.

The improvement and stabilization of balance during walking were the first step. His clinical status for recovery was that only walking was meaningful for him. Therefore, the therapeutic plans for the patient were as follows: (a) strengthening of standing‐related muscles with the correction of posture for stability during stance, and (b) reduction of gait freezing and festination and lengthening of stride for linear movement, including forward, backward, and side‐to‐side gait.

As a second step, training of the healthy normal stepping method, strengthening of the lower limbs with tango gait, and increasing variability of movement with music as cognitive therapy was targeted. Most noticeably, he showed typical Parkinsonian gait with freezing and festination. Characteristically, the patient showed a difference in stride length between the right side and the left side (Figure 3G,H).

Tango therapy was conducted for 1 hour per day, 5 days a week, for two weeks. After tango therapy for 10 hours in two weeks, the UPDRS score improved dramatically from 105 (grade 4) to 82 (grade 3) points, and the BBS score improved from 2 to 12 points (Table 1B).

His posture was improved dramatically to an upright posture (Figure 3A,B). His stepping pattern was slightly improved (Figure 3C,D). Although it was not a healthy stepping pattern, the weight‐bearing point moved to the heel with the upright correction of his posture (Figure 3D). His gait was also improved. The typical Parkinsonian gait was dramatically improved (Figure 3E,F), but the freezing and festination symptoms were felt by a therapist when walking with the patient and holding his elbow. His stride became wider; it was less than half of the length of the left foot before tango therapy, and it improved to nearly the length of the left foot after tango therapy (Figure 3I,J). The freezing symptom was improved but still evident. However, the festination was dramatically improved with greater stability during walking.

Tango therapy is dramatically effective for patients with PD with grave clinical severity as well as moderate severity.

The agreement of the two patients was obtained with a signed consent form and a signed permission form for photograph use, and this study was approved by the Institutional Review Board of Cheju Halla General Hospital (CHH2O19‐L01‐01).

## 3 DISCUSSION

Exercise therapy is an essential treatment for PD in addition to medical treatment and surgical treatment. However, there is no way to halt or stop the progression of the disease.

It may be necessary to explain the reason why the tango is appropriate for the therapy of PD and answer the following questions: Other than dance, what else is involved in tango therapy? What is the therapeutic strategy used to maximize the effectiveness of tango therapy for PD?

Argentine tango was reported to be more effective than other forms of dance as dance therapy for PD.^15^ From our results, Argentine tango is a suitable modality for rehabilitation of patients with PD. (a) Tango is possible with a safe, slow speed. (b) The range of movements is variable. However, tango movements are mostly within the range of natural movements. (c) Most frequently, tango gait has the effects of running, squatting, or climbing a mountain.^9^ The most important point is that tango therapy is very safe and effective in older subjects or patients with a neurologic disease because tango therapy is conducted at a safe, slow speed with effects similar to exercise. Therefore, tango is suitable as a therapy for PD, as well as other neurologic diseases and old age.

Tango therapy has good patient compliance because tango therapy is performed with music, and patients move according to the music. In particular, at the point of tango therapy where the patient adapts to the music beat, tango therapy is an appropriate therapeutic modality to correct the characteristics of Parkinsonian gait with freezing and festination because the patient is trained or forced to adjust to the regular beat of the music. Additionally, tango therapy is accompanied by music and has the effects of music therapy. Therefore, tango therapy is more than a dance in these aspects.

Tango therapy is also a physical therapy because tango movements consist of multiple elemental tango movements in which most body muscles are involved. Therefore, tango therapy has the effects of multiple physical therapy approaches performed simultaneously on the whole body, which are not able to be obtained in any other current physical therapy.

The difference of our tango therapy from other forms of tango therapy that have been performed is the application of tango movements element by element according to the clinical status of the patient.

The clinical statuses of the patients were analyzed by a tango therapist for the application of tango elements for tango therapy. First, the capability to stand upright was determined with the posture analysis. Then, the capability to walk was also analyzed. The step width and stride length were also measured for therapeutic planning to apply tango movements.^9^ Then, the therapeutic plans were made with the possible application of the basic elements and the movements of tango. The degree of movement was extended, and the tango movements were gradually added. The elemental application of tango movements became possible after the listing of basic tango elements^7^ and the functional anatomical analysis of these tango elements.^8^, ^9^, ^10^ Namely, it was proven that elemental clinical analyses and therapeutic plans are possible through this study.

The application of tango therapy began with the holding method. Holding of the palm seemed to be risky because patients had the possibility of falling down due to an unstable stance and gait.

The direct effects of tango therapy were the improvement and strengthening of stance, posture, and gait.^8^ These effects are essential for the clinical improvement of patients with PD, and they create dramatic psychological changes in the daily living of patients including improved self‐confidence. Additionally, the patients received psychological support with the therapist's attention to the patients during tango therapy.

In contrast to healthy subjects in which any elements are able to promote health, patients with PD have limited capabilities to stand and walk, and these are the first aspects that the tango therapist should understand and address. Of course, patients with severe PD were not able to receive tango therapy alone (solo tango was impossible) and were totally dependent on tango therapists. The independent solo tango was tried when the therapist was confident that the patients improved, and it was safe to do independently.

By involving patients with moderate to severe cases, all spectra of the sequences, purposes, and relevant methods used in tango therapy were systemically obtained. In the severe case, the therapeutic purpose and plan were established for patients with severe to moderate clinical status. In the moderate case, the therapeutic purpose and plan were established for patients with moderate to mild or nearly normal clinical status.

In the severe case, the first aim was to attain stance and gait regardless of whether it was dependent or independent. The second aim was to attain independent stance and gait. The third aim was to strengthen stance and gait according to the theory of tango therapy. Thereafter, cognitive therapy was applied by applying tango choreography, and music therapy was applied with a free tango salon. Finally, the strengthening of all of these aspects was able to be targeted.

Posture correction should always be kept in mind during tango therapy because posture affects the effectiveness of tango therapy. In the severe case, attaining a stable forward and backward gait was the main goal of training. With improvement, the side‐to‐side gait and posture correction were introduced.

However, in the moderate case, the training of side‐to‐side gait was performed, and thereafter, unique tango movements were added, including baldosa, which consisted of the forward, backward, and side‐to‐side gait.^6^ Salida normal and salida cruzada were also introduced after stance training with both feet crossed (Figure 2B).^6^ Accomplishment of these movements led to improved stability and strength of the lower extremities during the simple gait.

In the severe case, the length of the stride was very short, and the lengthening of the stride was the focus of training. Standing with both feet crossed and doing the salida normal and salida cruzada were impossible without a sufficient stride length.

The mood of the music was very important, and the therapist should pay attention to the selection of music. Additionally, the speed of the music was controlled according to the clinical status of the patient. If the patient showed improvement, tango music with a more rapid beat was chosen to increase the gait velocity of the patient for therapeutic purposes. Particularly, adjusting gait to a regular beat was very effective to correct the Parkinsonian gait with freezing and festination. Moreover, the self‐variation of movement speed was tried even with Parkinsonian gait symptoms after the improvement of gait.

In the moderate case, choreography was tried for the purpose of cognitive therapy (Figure 2D), and tango salon was tried for the effects of music therapy. When dancing tango with other people and exchanging gestures according to the tango music, the patient said that she was happy (Figure 2A).

Consequently, the tango therapy expert had a different perspective than the physician when approaching the patients for the effectiveness of therapy. The analysis of stance, posture, and gait by the tango therapy expert was more delicate than that in the clinical environment. Therefore, the therapeutic approach was also more precise and effective than classic physical therapy. Safety was the most important aspect of tango therapy because the movements in tango therapy were variable.

In patients with moderate to severe cases of PD, the delicate aspects of tango therapy, including medial arch stepping and pinching in tango stance^8^ and the reduction of step width, step angle, and center of mass (CoM) displacement in tango gait,^9^ were not considered due to the existence of higher priorities, such as a stable stance and gait. Therefore, the delicate aspects may be considered in patients with mild or suspected cases of PD to prevent the progression of the disease and apparent Parkinson's symptoms and promote the patient's health.

From this study, it was proven that the functional anatomical analysis of tango posture, stance, and gait with tango therapy was appropriate therapy for patients with PD^8^, ^9^, ^10^; from these analyses, the systemic analysis of the patient's clinical status and therapeutic plan for tango therapy was also appropriate for patients with PD. Through the systemic analysis and application of basic tango elements, tango therapy is very effective. Moreover, tango therapy is neither a simple physical therapy nor a simple dance. Until now, the benefits of tango therapy were generally suspected to be psychological in nature. On the contrary, the goal of tango therapy had been focused on the physical improvement in the medical reports. The most urgent medical problems of PD are the neuromuscular dysfunction. In this report, the 1st purpose and resultant apparent effects of tango therapy were improvement of neuromuscular impairment. However, tango therapy was reported to have psychologically beneficial effects and also had been applied in psychiatric fields. Tango therapy has both the physical and psychological effects for any disease, and tango therapy is more than just tango.

## 4 CONCLUSION

Based on these case studies, tango therapy is an effective treatment for PD. In the present study, we present an original approach of elemental tango therapy in two PD cases. Rush elemental tango therapy is an effective treatment for PD for only 2 weeks. Elemental tango therapy is appropriate for a systemic treatment, which can be designed specifically according to the clinical status of the patient using basic tango elements to strengthen the patient's disabilities. Tango therapy is very suitable for patients with neurologic diseases and older subjects because it is safe due to the ability to perform the movements at a slow speed and has effective exercise effects due to the range of movements being within the natural and proper range of weighted movements without the loss of balance. Tango therapy is a combination therapy of multiple physical therapy approaches with the addition of music therapy and cognitive therapy. However, the effects of tango therapy seem to be more than the summation of multiple physical therapy approaches.

## CONFLICT OF INTEREST

The authors declare that there is no conflict of interest regarding the publication of this manuscript.

## AUTHOR CONTRIBUTIONS

YK: is the main author of this manuscript. GN: coordinated all processes of this study.

## Supporting information

VideoS1
